# Chemical profile of *Rhododendron luteum* Sweet leaf supercritical CO_2_ extracts and their anti-inflammatory and antidiabetic potential

**DOI:** 10.3389/fchem.2025.1576852

**Published:** 2025-04-25

**Authors:** Lena Łyko, Renata Nowak, Marta Olech, Urszula Gawlik, Agnieszka Krajewska, Danuta Kalemba, Andriy Prokopiv

**Affiliations:** ^1^ Department of Pharmaceutical Botany, Medical University of Lublin, Lublin, Poland; ^2^ Department of Biochemistry and Food Chemistry, University of Life Sciences, Lublin, Poland; ^3^ Institute of Natural Products and Cosmetics, Lodz University of Technology, Łódź, Poland; ^4^ Department of Botany, Botanical Garden, Ivan Franko National University of Lviv, Lviv, Ukraine

**Keywords:** *Rhododendron luteum*, LC-MS, terpenes, β-sitosterol, flavonoids, antiinflammatory, antidiabetic, SFE

## Abstract

**Introduction:**

Terpenes are a diverse class of natural metabolites that exhibit a range of biological activities, including antidiabetic, antiproliferative, and anti-inflammatory effects. The present study was designed to determine the chemical profile and biological activity of *Rhododendron luteum* leaf (RLL) supercritical CO_2_ (SC-CO2) extracts.

**Materials and methods:**

The LC-APCI-MS/MS method was used to determine the composition and content of the triterpenes (including sterols). In turn, LC-ESI-MS/MS was applied for the analysis of polyphenolic compounds. The volatile composition of RLL extracts was analysed using the HS-SPME-GC-FID-MS technique. The inhibitory activity against enzymes such as xanthine oxidase (XO), lipoxygenase (LOX), hyaluronidase, α-amylase, and α-glucosidase was assessed using *in vitro* bioassays.

**Results and discussion:**

The LC-MS analyses revealed high levels of oleanolic acid, ursolic acid, and β-sitosterol, which belong to terpenes, as well as polyphenols such as syringic acid, ferulic acid, and p-coumaric acid, along with apigenin and its 7-glucoside. Among volatiles, the most prominent were limonene, eugenol, β-phenylethanol, and β-caryophyllene. *In vitro* assays showed a high hyaluronidase and moderate lipoxygenase and xanthine oxidase inhibitory activity presented by RLL supercritical extracts. Moreover, the samples were found to present α-glucosidase inhibitory activity. This may indicate the anti-inflammatory and antidiabetic potential of RLL supercritical extracts.

## 1 Introduction

*Rhododendron* is the most numerous genus in the Ericaceae family. It is spread in the Northern Hemisphere in Asia, North America, and Europe. According to ethnobotanical data, plants of this genus have been used to treat various diseases for hundreds of years (Popescu and Kopp, 2013). New scientific reports confirm their activity and indicate the specific secondary metabolites responsible for this action. The main chemical constituents of this genus were found to be phenolics, especially flavonoids, and terpenes, including triterpenes (Qiang, Zhou, and Gao, 2011; Grimbs et al., 2017; Liu et al., 2024).

One of the *Rhododendron* species is *Rhododendron luteum* Sweet (RL; syn. *Rhododendron flavum*; commonly known as the yellow azalea or the honeysuckle azalea). It occurs naturally at different geographical latitudes. The species is still poorly explored. Previous studies mainly analysed the composition of *R. luteum* polyphenols (Mahomoodally et al.,et al., 2020; Şeker and Eedogan, 2023; Yeşil and Akgül, 2023). There are also reports characterizing the composition of monosaccharides, fatty acids, carotenoids, and volatile compounds (Tasdemir et al., 2003; Carballeira et al., 2008; Usta et al., 2012; Yeşil and Akgül, 2023). Conversely, research is scarce on the composition of terpenes. In turn, the biological activity of *R. luteum* extracts has been the subject of slightly more studies. Research on specimens growing in Turkey has shown anticancer potential, as well as the ability to inhibit enzymes involved in the pathogenesis of Alzheimer’s disease and diabetes (Demir, Turan, and Aliyazicioglu, 2018; Mahomoodally 2020; Turan et al., 2021). Our previous research on the yellow azalea of Polish origin has focused on investigating RL leaf antioxidant and anti-inflammatory activity. It demonstrated the ability of the extracts to inhibit enzymes such as lipoxygenase, cyclooxygenase, and hyaluronidase (Olech et al., 2020; Łyko et al., 2022).

Supercritical CO2 (SC-CO2) extraction allows for selective elution of specific secondary metabolites from rich plant matrices, depending on parameters such as pressure and temperature. The use of carbon dioxide in SC-CO_2_ extraction has many advantages. It is safe, non-toxic and easily available (Wrona et al., 2017). This method effectively extracts non-polar compounds and can be adjusted for more polar compounds by adding non-toxic co-solvents, such as water or ethanol (Domingues et al.,et al.,et al., 2012; Verónico Sánchez et al., 2020; Łyko et al., 2024). Since SC-CO works at low temperatures, it preserves labile bioactive compounds such as flavonoids, triterpenes, and carotenoids. This is a significant advantage over traditional methods such as Soxhlet extraction, infusion, or ultrasound-assisted extraction, where higher temperatures or ultrasounds may degrade compounds (Domingues 2012; Tyskiewicz, Konkol, and Rój, 2018). This new method is gaining in popularity due to its low environmental impact. What is more, it helps to avoid environmentally harmful extraction auxiliaries, such as toxic and volatile organic solvents (Domingues 2012). On the other hand, SC-CO_2_ extraction may be more complex in optimization since fine-tuning parameters (pressure, temperature, and particle size) are more challenging. Moreover, it requires a high initial investment due to the need for specialized equipment to handle the high pressure and temperature involved (Lang and Wai, 2001).

Our latest research on the composition and bioactivity of RL supercritical flower extracts allowed us to identify and determine new valuable metabolites (Łyko et al., 2024). Some of the identified compounds would probably not elute or elute much less if classical extraction methods were used. Therefore, this study is devoted to the analysis of terpenes, phytosterols, phenolic acids, flavonoids, and volatile compounds, as well as the pharmaceutical potential of *R. luteum* leaf supercritical extracts. Two samples with different polarities were obtained and subjected to analysis of the composition of triterpenes and phytosterols with the use of LC-APCI-MS/MS (liquid chromatography/atmospheric pressure chemical ionisation-triple quadrupole mass spectrometry). Both samples were also tested to determine the content of phenolic acids and flavonoids using LC-ESI-MS/MS (liquid chromatography/electrospray ionisation-triple quadrupole mass spectrometry). Additionally, volatiles in a non-polar sample were investigated using HS-SPME-GC-FID-MS (headspace solid-phase microextraction, followed by gas chromatography with flame ionisation detection coupled with mass spectrometry). Both extracts were studied for their anti-inflammatory and antidiabetic effects. Moreover, the potential of supercritical extracts to inhibit relevant enzymes, such as lipoxygenase, hyaluronidase xanthine oxidase, α-amylase, and α-glycosidase, was determined.

## 2 Materials and methods

### 2.1 Chemicals and apparatus

Analytical standards such as lupeol and ursolic acid were obtained from Chromadex (Irvine, CA, United States). Hyperoside, prunetin, α-amyrin, β-sitosterol, stigmasterol, maslinic acid, oleanolic acid, isoquercetin, apigetrin, luteoloside, syringic acid, 5-O-caffeoylquinic acid, gallic acid, protocatechuic acid, salicylic acid, p-coumaric acid, ferulic acid, as well as soybean 15-lipooxygenase, linoleic acid, xanthine oxidase, allopurinol, quercetin, bovine serum albumin, hyaluronidase, hyaluronic acid, sodium phosphate monobasic solution, sodium phosphate dibasic solution, sodium chloride solution, sodium acetate, acetic acid, starch, 3,5-dinitro-2-hydroxybenzoic acid (DNS reagent), acarbose, α-amylase, α-glucosidase, sodium carbonate, p-nitrophenyl-α-D-glucopyranoside (pNPG), HPLC and LC-MS grade methanol and acetonitrile were supplied by Sigma-Aldrich Fine Chemicals (St. Louis, MO, United States). Chloroform and phosphate buffer were from Avantor Performance Materials Poland S.A. (Gliwice, Poland). Apigenin was sourced from Roth (Karlsruhe, Germany).

Spectrophotometric analyses were performed with the Infinite Pro 200 F micro-plate reader (Tecan Group Ltd.; Männedorf, Switzerland) with the use of transparent/black 96-well microplates (Nunclon, Nunc; Roskilde, Denmark). The Millipore Direct-Q3 purification system (Bedford, MA, United States) was used to obtain HPLC-grade water. Samples were evaporated with the Heidolph Basis Hei-VAP Value evaporator (Schwabach, Germany). The Zone 1 apparatus (Labconco, Kansas City, KS, United States) was used for lyophilization.

### 2.2 Plant material

*Rhododendron luteum* Sweet leaves were collected in the Zhytomyr Region near Olevsk (51°13′43.96″N 27°37′18.20″E; Ukraine) in July 2021. The material was dried at ambient temperature and deposited at the Department of Pharmaceutical Botany, Medical University of Lublin, Poland (voucher No. RL-03/21). Before extraction, the leaves were ground and sifted through a sieve. Grains with a size under ø1.5 mm were selected for the extraction.

### 2.3 Supercritical CO_2_ extraction

Supercritical fluid extraction of *R. luteum* leaves was performed using a laboratory scale installation with supercritical carbon dioxide at the Łukasiewicz Research Network–New Chemical Syntheses Institute in Puławy (Poland). The dynamic supercritical fluid extractor worked at temperatures up to 80°C and pressures up to 450 bar. The extraction process of RL material was previously described (Łyko et al., 2024). In brief, two extraction stages were conducted at 40°C–50°C and 300 bar. The non-polar sample was obtained using pure carbon dioxide; the second (more polar) sample was obtained due to the addition of 30% aq. Ethanol as a co-solvent (Wrona et al., 2017).

### 2.4 LC-APCI-MS/MS analysis of triterpenes and polyphenols

The LC-MS/MS system comprised an Agilent 1,200 Series chromatograph (Agilent Technologies, Santa Clara, CA, United States) paired with a 3200 QTRAP linear ion trap quadrupole mass spectrometer, which featured a Turbo V™ source (Sciex, Redwood City, CA, United States). Separations of polyphenols were performed on an Eclipse XDB-C18 analytical column (4.6 × 150 mm, 5 μm; Agilent Technologies, United States). The chromatographic conditions are given in Supplementary Tables S1, S2. The 3200 QTRAP mass spectrometer worked in the negative ion mode, using multiple reaction monitoring (MRM) mode. An electrospray ionisation (ESI) was used for the polyphenolic profile analysis. Optimal mass analyser settings - including MRM transitions, declustering potential, entrance potential, collision cell exit potential, collision energy, and product ion selection - were experimentally determined for each compound. Instrument parameters included an ion spray voltage of −4,500 V, a temperature of 500°C, CUR gas at 23 psi, and nebuliser (gas1) and heater (gas2) pressures of 50 and 60 psi, respectively. Before LC injection, the samples were filtered through a 0.20 µm hydrophilic polytetrafluoroethylene (PTFE) syringe filter (Merck, Darmstadt, Germany).

For the triterpene analysis, the RL samples were separated on the Kinetex XB-C18 column (150 × 2.1 mm; particle size 2.6 μm, Phenomenex, Torrance, CA, United States). The detailed separation conditions are given in our previous paper (Łyko et al., 2024). In brief, the gradient elution with 0.1% formic acid in water and 0.1% formic acid in ACN at 35°C was used. The flow rate was 350 μL/min, and the injection volume was 5 μL. An atmospheric pressure chemical ionisation (APCI) source was working in both positive and negative modes. The instrument parameters for APCI (−) included: collision gas set at 3, nebuliser current at −5, curtain gas at 20 psi, temperature at 450°C, and ion source gas at 25 psi. For APCI (+), the settings were: collision gas at 4, nebuliser current at 4, curtain gas at 30 psi, temperature at 300°C, and ion source gas at 35 psi. Analytes were detected and quantified using the multiple reaction monitoring (MRM) mode. The most intense MRM transitions, along with their optimal parameters, were experimentally determined for each compound. MS data were acquired and processed using Analyst 1.5 software (AB Sciex, Redwood City, CA, United States). Quantification was based on the peak areas of the most intense MRM transitions, using calibration curves generated for each standard (see Supplementary Table S2 in Supplementary Materials). The limit of detection (LOD) and limit of quantification (LOQ) were set at signal-to-noise ratios of 5:1 and 10:1, respectively. Each standard solution and all the samples were assayed in triplicate.

### 2.5 HS-SPME-GC-MS/FID analysis of volatiles

For the analysis of volatiles from *R. luteum* supercritical CO_2_ extracts, the HS-SPME-GC-FID-MS technique was employed. Sampling was performed using SPME grey fiber 50/30 μm DVB/CAR/PDMS, stableflex 2 cm (Supelco Bellefonte, United States). RLL-CO2 sample (25 mg) was placed in a 15 mL Amber vial with hole cap PTFE/Silicone Septa (Supelco Bellefonte, United States). Each sample was incubated at 60°C for 30 min before extraction. Then, the fibre was introduced into the vial, and a 30-minute absorption was carried out. The fibre was desorbed directly in GC-FID-MS for 10 min, with the analysis conducted in two repetitions. A Trace GC Ultra gas chromatograph, coupled with a DSQ II mass spectrometer (Thermo Electron Corporation), was utilized for the analysis. A simultaneous GC-FID and MS analysis was achieved using an MS-FID splitter (SGE, Analytical Science, Austin, TX, United States). The operating conditions were as follows: a non-polar capillary column Rtx-1ms (60 m × 0.25 mm, 0.25 μm film thickness) was used, with a temperature program of 50°C (held for 3 min) to 300°C at a rate of 4°C/min. The injector (SSL) temperature was set at 280°C, the FID detector at 300°C, and the transfer line at 250°C. Helium served as the carrier gas, maintaining a constant pressure flow of 200 kPa with a split ratio of 1:20. The mass spectrometer settings included an ion source temperature of 200°C, ionisation energy of 70 eV (EI), operating in full scan mode with a mass range of 33–420. Component percentages were calculated from the GC peak areas without applying a correction factor. Component identification was based on a comparison of their mass spectra and linear retention indices (RI, non-polar column), determined against a series of n-alkanes (C8-C24) and literature data along with computer databases NIST 2011 and MassFinder 4.1 (Adams and Sparkman, 2007).

### 2.6 Xanthine oxidase inhibition assay (XO)

The xanthine oxidase inhibition activity was determined according to the method described by Pieczykolan et al. (2021). Basically, the extract (40 µL) was mixed with the phosphate buffer (100 μL; 0.066 M, pH 7.8) and enzyme (20 μL; 0.01 U/mL), and incubated for 10 min. After this time, the reaction started with the addition of the substrate (115 μL; 0.44 mg/mL). The absorbance increase was monitored at λ = 295 nm over a 2-minute period. Allopurinol was used as a positive control, and 50% ethanol was used as a blank. All measurements were made in triplicate. The ability of the sample to inhibit the enzyme is expressed as IC_50_ values, which were calculated based on the dose-response curves. The mode of inhibition of the enzyme was established with the use of the Lineweaver–Burk plots.

### 2.7 Lipoxygenase inhibition assay (LOX)

The inhibition of lipoxygenase was measured spectrophotometrically (Habza-Kowalska et al., 2019). Basically, the absorbance (λ = 234 nm) of the mixture containing sample (10 µL), phosphate buffer (240 μL; 0.066 M; pH 7.8), and enzyme (10 μL; 167 U/mL) was monitored after linoleic acid addition (40 μL; 2.5 mM of linoleic acid). The positive control was quercetin, while 50% ethanol was used as a blank. All measurements were made in three replicates. The lipoxygenase inhibition activity was expressed as IC_50_ values. These were calculated based on dose-response curves, which were plotted with the use of different concentrations of RL extracts and positive control. The enzyme inhibition mode was performed using the Lineweaver–Burk plots.

### 2.8 Hyaluronidase inhibition assay

The assay of hyaluronidase inhibitory activity was conducted as previously described by Łyko et al. (2022). In brief, 20 µL of the sample or the positive control was mixed with 20 µL of the hyaluronidase enzyme (0.1 mg/mL). After 10 min of incubation at 37°C, 20 µL of hyaluronic acid (0.5 mg/mL) was added. After incubation (45 min at 37°C), the reaction has been stopped with 100 µL of acidic albumin solution. Then, it was incubated for another 10 min at room temperature. After this time, the transmittance was measured at 600 nm wavelength. All of the measurements were made at least in triplicate. The rate of enzyme inhibition was calculated in the same way as in the previous study (Łyko et al., 2022). Based on the measurements of different sample concentrations, the dose-dependent curve was plotted, and the IC_50_ values were determined.

### 2.9 α-glucosidase inhibition assay

The α-glucosidase inhibitory activity of the extracts was evaluated using the method previously described by Pieczykolan et al. (2021). In this assay, a mixture consisting of 10 µL of the α-glucosidase enzyme (1 U/mL in 0.1 M phosphate buffer at pH = 6.8), 50 µL of 0.1 M phosphate buffer (pH 6.8), and 20 µL of the sample at different concentrations was prepared in a 96-well microplate. This mixture was incubated at 37°C for 15 min. To initiate the enzymatic reaction, 20 µL of a 5 mM p-nitrophenyl-α-D-glucopyranoside solution in phosphate buffer was added, and the plate was incubated for another 20 min at 37°C. The reaction was stopped by adding 50 µL of 0.1 M sodium carbonate. The absorbance was read at 405 nm using a microplate reader. Controls included a reaction system without the plant extracts and a blank without the enzyme to account for background absorbance.

### 2.10 α-amylase inhibition assay

The α-amylase inhibition assay followed the method outlined by Pieczykolan et al. (2021). For this assay, 100 µL of the sample was mixed with 100 µL of 0.02 M sodium phosphate buffer (pH 6.9) and 100 µL of an α-amylase solution (4.5 U/mL) and pre-incubated at 25°C for 10 min. Subsequently, 100 µL of a 1% starch solution was added, and the mixture was incubated at 25°C for 30 min. To stop the reaction, 1 mL of a DNS reagent was added, followed by incubation in a boiling water bath for 5 min, after which the mixture was cooled to room temperature. The final solution was diluted 10-fold with distilled water, and the absorbance was measured at 540 nm. A control sample (with a buffer instead of the plant extract) was used for comparison.

### 2.11 Statistical analysis

All tests were performed at least in triplicate, and the results are expressed as a mean with standard deviation (SD). Excel was used to conduct statistical analyses.

## 3 Results and discussion

### 3.1 Supercritical CO_2_ extraction of RL leaves

Supercritical extracts of *R. luteum* leaves were obtained using a laboratory-scale installation, previously described for the RL flowers method (Łyko et al., 2024). In brief, to elute both non-polar metabolites (e.g., volatiles and phytosterols) and more polar terpenes (e.g., triterpenic acids) and polyphenols, the process was divided into two steps. The first one was performed with pure CO_2_ and the second with CO2 and the addition of 30% aqueous ethanol. The 70/30 aqueous ethanol was used instead of azeotropic ethanol to grow the elution of more polar compounds. The extraction efficiency of the first step was 3.15% while adding aqueous ethanol as a co-solvent in the subsequent step increased the yield to 26.76%. A similar situation was observed in our early study of RL flowers (Łyko et al., 2024).

The main aim of the study was to evaluate the terpenes and phytosterol profile of RL leaves in the context of their biological activity. For this purpose, SFE extraction with carbon dioxide was chosen for the first time for this plant material. The preliminary operating conditions for the SFE extraction of non-polar compounds from RL leaves were experimentally selected and according to literature data (Herrero et al., 2010; Domingues et al., 2012; Tyskiewicz et al., 2018).

We decided to use the high-pressure value, preferring elution of a wider range of non-polar compounds, especially terpenes, phytosterols, and volatiles. The increase in the extraction temperature to 50°C and the ethanol addition, in the second stage of extraction, were applied to increase the elution of more polar compounds (from groups of terpenes and phenols) from plant material. In the second stage of supercritical CO_2_ extraction (performed at high pressure, 300 bar), the addition of 30% ethanol enhances the polarity and selectivity of the supercritical fluid solvent, connected with its properties (e.g., low viscosity, high density, and diffusivity) which promote the elution of a wide range of compounds, including lipophilic substances with higher polarity (such as triterpene acids and alcohols, glycosidic forms), and less-polar polyphenolic compounds, such as phenolic acids, flavonoid aglycones, and some phenolic glycosides.

Our previous research on *R. luteum* leaves has focused on the polyphenol composition in polar extract obtained using two-step classical extraction (Łyko et al., 2022). In the cited article, RL leaves were first pretreated with petroleum and chloroform in a Soxhlet apparatus to remove the ballast (in this case) lipophilic substances, and then it was extracted with alcohol to obtain a polyphenol-rich sample (in the second stage).

In contrast to SC-CO2, the second extraction stage in classical maceration (at low temperature, 25°C, and atmospheric pressure, approx. 1 bar) with aqueous ethanol is commonly employed in phytochemical analyses (Łyko et al., 2022). The elution ability of this solvent is somewhat limited to the elution of more polar and hydrophilic compounds, such as phenolic acids, flavonoid glycosides, and carbohydrates. Furthermore, this approach is more time-consuming, although it can be optimized by incorporating ultrasound-assisted extraction or by increasing the ethanol concentration (e.g., to 80%), which is often used for a broad range of polar compounds (Olech et al., 2020).

The composition and biological activity of the extracts obtained by these two distinct methods will differ considerably. Therefore, further research and optimization of extraction methods are necessary to get the most biologically active and rich composition extracts. Our exhaustive study of the composition of RL leaves shows that to obtain a high yield of polyphenols, it would be necessary to use the classic method with aqueous alcohols or the ASE method, while in order to extract as many terpenes as possible, SC-CO2 will work much better.

### 3.2 Terpene and phytosterol content in *Rhododendron luteum* leaf supercritical extracts

Pentacyclic triterpenes are a diverse class of natural products that exhibit a range of biological activities. Among others, they have been proven to have anti-inflammatory, antiviral, antidiabetic, and antitumor potential (Hernández-Vázquez et al., 2012). Our previous studies have revealed a high content of triterpene compounds in RL flowers (Łyko et al., 2024). The content of triterpenes in RL leaves has not yet been fully investigated. To the best of our knowledge, the last research on triterpenes in RL dates back to the 1980s and is quite modest (Belova and Fokina, 1970; Fokina, 1980).

The triterpene profile of the *R. luteum* leaf SC-CO2 extract was determined using the LC-APCI-MS/MS method in both positive and negative atmospheric pressure chemical ionisation (APCI) modes. The components were detected and quantified using the multiple reaction monitoring (MRM) mode. The study resulted in the detection and quantification of pentacyclic triterpenes, such as maslinic acid, corosolic acid, ursolic acid, oleanolic acid, erythrodiol, uvaol, lupeol, 3β-taraxerol, and α-amyrin, as well as β-sitosterol belonging to phytosterols (Table 1).

**TABLE 1:** Triterpene content (mg/g of dry extract) in *Rhododendron luteum* Sweet leaf extracts.

Constituent	RLL -CO_2_	RLL-CO_2_+30%EtOH
β-Sitosterol	16.95 ± 0.35	26.80 ± 0.52
3β-Taraxerol	17.50 ± 0.14	13.30 ± 0.14
α-Amyrin	5.97 ± 0.02	5.36 ± 0.06
Lupeol	1.96 ± 0.03	1.61 ± 0.01
Erythrodiol & Uvaol	2.91 ± 0.04	5.00 ± 0.05
Oleanolic & Ursolic acid	7.00 ± 0.02	145.33 ± 5.86
Corosolic acid	0.32 ± 0.00	3.32 ± 0.01
Maslinic acid	1.40 ± 0.01	12.45 ± 0.49

Abbreviations: RLL-CO_2_, *R. luteum* leaf supercritical extract obtained with pure CO_2_, RLL-CO_2_+30%EtOH–RL, supercritical leaf extract obtained with the addition of 30% ethanol; BQL, compound detected with a concentration below the quantification limit.

The RLL-CO2 sample obtained with pure CO_2_ contained lower concentrations of most analysed metabolites. 3β-taraxerol and β-sitosterol (17.50 ± 0.14 and 16.95 ± 0.35 mg/g of DE, respectively) were found to be its predominant metabolites. The concentration of these metabolites was lower than we had observed in the RL flower supercritical extract (Łyko et al., 2024). Taraxerol was also detected in leafy shoots of RL growing in the territory of the former Soviet Union (Belova and Fokina, 1970). More polar molecules (i.e., erythrodiol, uvaol, and oleanolic, ursolic, corosolic, and maslinic acids) were previously observed in comparable amounts in our research of the *R. luteum* flower SC-CO2 extract (Łyko et al., 2024). They were also found in other rhododendron species. For example, erythrodiol was detected in *Rhododendron concinnum* and *Rhododendron collectianum*, maslinic acid occurred in *Rhododendron anthopogonoides*, α-amyrin was found in *Rhododendron brachycarpum*, lupeol was observed in the bark of *Rhododendron arboreum*, and uvaol was present in *Rhododendron ellipticum* (Qiang et al., 2011; Liu et al., 2024).

Ethanol used as a co-solvent in the second stage of the SC-CO2 extraction procedure resulted in much higher yields of observed compounds. However, the content of non-polar compounds (i.e., β-sitosterol, 3β-taraxerol) in both extracts was comparable, the RLL-CO2+30%EtOH sample contained much higher amounts of polar constituents, e.g., maslinic, oleanolic, corosolic, ursolic acid, erythrodiol, and uvaol.

We have tested numerous mobile phase gradients and other chromatographic conditions. However, due to a very similar structure, m/z of the precursor ion, and fragmentation pattern, we could not achieve a proper separation of erythrodiol and uvaol (Łyko et al., 2024). Thus, we decided to present their content as a sum of erythrodiol and uvaol based on the erythrodiol calibration curve. A re-extraction of the material with the addition of a polar co-solvent resulted in the release of significant amounts of triterpene acids, particularly oleanolic and ursolic acids (145.33 ± 5.86 mg/g DE). These molecules were present in RLL-CO2+30%EtOH in much greater amounts than in the corresponding RL flower extract (Łyko et al., 2024). In the case of these compounds (similarly to uvaol and erythrodiol), the separation was not satisfactory, and the result was provided as a sum of the compounds based on the oleanolic acid calibration curve. These secondary metabolites present multiple pharmacological effects. Among others, they have anticancer, antidiabetic, anti-infectious, lipidemic, cardioprotective, antiosteoporotic, anti-inflammatory, and neuroprotective properties (Mioc, 2022a; Zhao et al., 2023) Moreover, both compounds are considered non-toxic and are used as ingredients in dietary supplements (Geerlofs et al., 2020; Ray et al., 2024). The other detected compound, maslinic acid, was reported to also has antidiabetic and anticancer effects. Antibacterial and antiviral activity was found as well (Mioc, 2022b).

### 3.3 Polyphenolic compounds of *Rhododendron luteum* leaf supercritical extracts

*R. luteum* supercritical extracts were additionally subjected to LC-ESI MS/MS (liquid chromatography electrospray ionisation mass spectrometry) analysis. As a result, phenolic acid, flavonoid aglycone, and flavonoid glycoside contents were determined in non-polar RLL-CO2 and more polar RLL-CO2+30%EtOH extracts.

As presented in Table 2, the non-polar extract contained trace amounts of polyphenols. The only phenolic compounds were ferulic acid (39.40 ± 3.80 μg/g of DE), isorhamnetin (67.67 ± 3.67 μg/g DE) and prunetin (26.60 ± 0.40 μg/g). Polyphenol elution was increased with the addition of co-solvent. The main polyphenol components in the RLL-CO2+30%EtOH extract were syringic, ferulic, and p-coumaric acids (9613.33 ± 114.70; 3850.00 ± 70.00; 1842.67 ± 103.09 μg/g of dry extract, respectively). Among all the detected phenolic acids, only syringic and sinapic acids have not been observed in RL to date (Mahomoodally et al., 2020; Olech et al., 2020; Łyko et al., 2022; Şeker and Eedogan, 2023). The amounts of dominant flavonoids were relatively lower–e.g., of apigenin (968.67 ± 1.89 μg/g DE) and its glycoside–apigetrin (948.00 ± 69.81 μg/g). However, these compounds are very promising in terms of their biological activity. Apigenin and apigetrin exhibit anti-cancer effects (Liao et al., 2023). Moreover, along with prunetin and luteolin, it shares structural similarities with isocumarins, well-known for their anti-inflammatory and anti-diabetic potential (Ramanan et al., 2016; Sudarshan and Aidhen, 2017). Syringic acid has shown promise in protecting against various oxidative stress-related diseases, type 2 diabetes, neurodegenerative diseases, cardiovascular diseases, and cancer (Bartel et al., 2024). Another compound, ferulic acid, demonstrates antioxidant, anticancer, anti-inflammatory, hepatoprotective, antimicrobial, and antiviral effects, while also influencing enzyme function (Pyrzynska, 2024).

**TABLE 2:** Polyphenol content (µg/g of dry extract) in *Rhododendron luteum* Sweet leaf extracts.

Constituent	RLL-CO_2_	RLL-CO_2_+30%EtOH
Gallic acid	—	107.87 ± 4.41
5-*O*-caffeoylquinic acid	—	15.51 ± 1.03
Gentisic acid	—	BQL
Syringic acid	—	9613.33 ± 114.70
p-Coumaric acid	—	1842.67 ± 103.09
Sinapic acid	—	394.00 ± 5.00
Salicylic acid	—	1,405.33 ± 10.50
Ferulic acid	39.40 ± 3.80	3850.00 ± 70.00
Isoferulic acid	—	554.00 ± 1.00
Protocatechuic acid	—	1,586.00 ± 38.00
Isorhamnetin	67.67 ± 3.67	242.67 ± 9.97
Luteolin	—	127.13 ± 3.18
Apigenin	—	968.67 ± 1.89
Prunetin	26.60 ± 0.40	208.60 ± 2.60
Hyperoside and isoquercetin	—	218.80 ± 2.80
Luteoloside	—	86.80 ± 5.00
Apigetrin	—	948.00 ± 69.81

Abbreviations as in Table 1; “-” – not detected.

Many scientific reports outline the high selectivity of SC-CO2 extraction for non-polar compounds (due to the non-polar nature of CO_2_) in comparison to other extraction methods, such as accelerated solvent extraction (ASE) or extraction in a Soxhlet apparatus (Wrona et al., 2017; Tyskiewicz et al., 2018; Tyskiewicz et al., 2019). However, this selectivity can be enhanced by adding co-solvents like ethanol or methanol to target more polar compounds, which is very noticeable in our research. The SC-CO2 extract is dominated by medium and weakly polar phenolic acids, which is quite the opposite of the RL extracts obtained by the ASE method and by maceration, where one of the most polar, 5-O-caffeoylquinic acids dominated (Olech et al., 2020; Łyko et al., 2022). Similarly, among the flavonoid aglycones noted in RL, the least polar compounds were observed in the highest amounts. Our study is in agreement with some other research comparing the polyphenol composition of the SC-CO2 extract to those obtained by conventional methods (Farías-Campomanes et al., 2015). In the cited paper, there were significant differences in polyphenolic profiles observed in Soxhlet extract and the SC-CO2 extract of lees. Another paper comparing the total polyphenol content (TPC) of SC-CO2 and ASE confirms the greater selectivity of SC-CO2 over ASE and, thus, the much lower TPC value (Rodríguez-Solana et al., 2015).

### 3.4 Volatiles of *Rhododendron luteum* leaf supercritical extract

The RLL-CO2 extract obtained in the first step of extraction was suspected to contain high amounts of volatile compounds. Therefore, *R. luteum* leaf supercritical CO_2_ extract was analysed by means of headspace solid-phase microextraction, followed by gas chromatography and flame ionisation detection coupled with mass spectrometry (HS-SPME-GC-FID-MS) to determine the content of volatile constituents (He et al., 2022). Since more volatiles were extracted in the first stage, and given that the use of polar modifiers (such as alcohol and water) in the second stage of extraction caused a decrease in the volatiles yield, it was decided not to analyse the RLL-CO2+30%EtOH extract in this regard (Pourmortazavi and Hajimirsadeghi, 2007).

**FIGURE 1:**
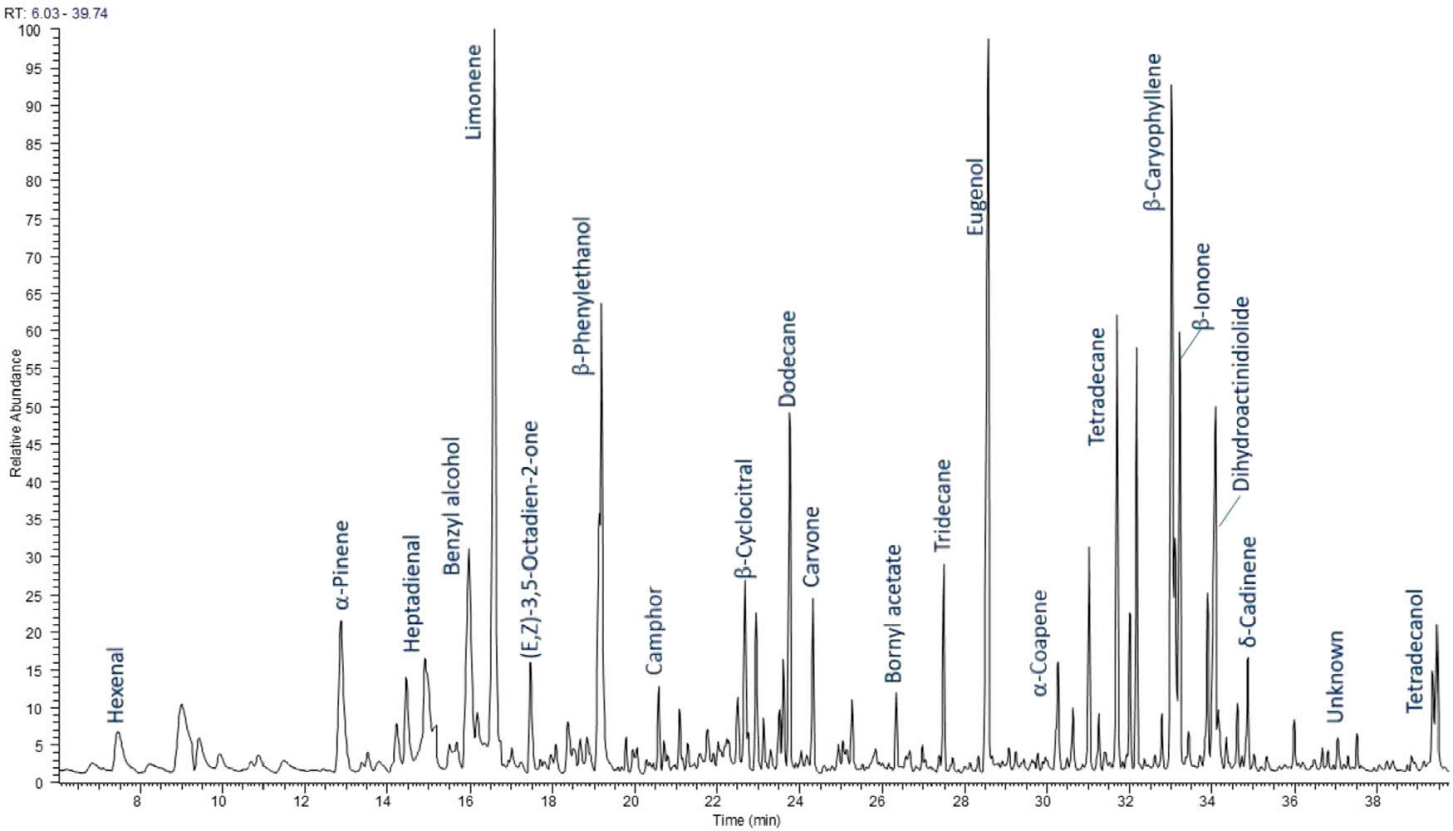
Chromatographic profile of volatile constituents of *R. luteum* leaves supercritical extract (RLL-CO2).

HS-SPME-GC-FID/MS analysis revealed over 100 compounds belonging to different groups of volatile metabolites (Supplementary Table S3 in Supplementary Materials). Most of them were monoterpene and sesquiterpene hydrocarbons and their oxygenated derivatives. Many aromatic and aliphatic compounds were also detected. The qualitative composition of volatiles in the RLL-CO2 extract was similar to that of the flower supercritical extract obtained from the same plant in our previous study (Łyko et al., 2024), although quantitative differences were observed. More than seventy compounds identified in leaves were present in the flower SC-CO2 extracts. The main common volatile constituents in both extracts were eugenol (6.15% and 7.26% in the leaf extract and flower extract, respectively), β-phenyl ethanol (5.22% and 5.20%), dodecane (5.58% and 5.07%), β-caryophyllene (3.91% and 3.09%), and dihydroactinidiolide (3.82% and 1.84%). Other important and dominant components of leaf volatiles were limonene (13.10%), nonanal (3.28%), and β-ionone (3.03%), present in the flower extract in amounts lower than 0.5%. The chromatographic profile of volatile constituents in *R. luteum* leaves SC-CO2 extract is shown in Figure 1.

These results differed significantly from those previously reported for RL collected in Turkey (Tasdemir et al., 2003; Alan et al., 2010; Usta et al., 2012; Gokturk and Burjanadze, 2023). It is worth noting that the study of Alan et al. (2010) concerned volatiles emitted by aerial parts of *R. luteum*, while other cited papers report on the essential oil isolated from the plant. The research most similar to ours was conducted by Tasdemir (2003). The authors analysed volatiles trapped by HS-SPME from above hexane and dichloromethane extracts of different *Rhododendron* species. Benzyl alcohol and β-phenylethanol, belonging to the main compounds in our research, were also found in abundance in the dichloromethane extract of *R. luteum* leaves (17.1%, 6.7%). These compounds were present in small amounts in the leaf hexane extract (1.6%, 1.2%) (Tasdemir 2003). Four other compounds found in the hexane extract in the amount higher than 5%, namely, 6-methylhept-5-en-2-one, undecane, α-terpineol, and oct-1-en-3-ol, were identified in our RLL-CO2 sample, but in smaller amounts. On the other hand, other significant components of dichloromethane extract, such as butanol (58,7%), 2-(2-ethoxy ethoxy) ethanol (3.3%), and γ-butyrolactone (2.9%) were not identified in the RLL-CO2 sample in our study (Tasdemir 2003). Noted in another study, the main volatiles emitted by aerial parts of the plant were hydrocarbons β-caryophyllene 34.0%), α-pinene (10.0%), and (E)-β-ocimene (10.4%), as well as methyl benzoate (11.7%) (Verónico Sánchez et al., 2020). It is worth mentioning that the compositions of essential oil distilled from *R. luteum* flowers collected in two localities in Turkey show some similarities and some differences between each other (Usta, 2012; Gokturk and Burjanadze, 2023), as compared with HS-SPME in the HS-SPME-GC-MS study on the leaf of the Turkish specimen (Tasdemir, 2003).

Hence, it is worth noting that the composition of volatile compounds in a given genus or species may differ significantly depending on the degree of phylogenetic relationship, the anatomical part of the plant being studied, or the geographical region. Other factors are also important, such as plant growth conditions, habitat, sunlight, amount of rainfall, microbial infections, and other plant species nearby. This may cause observed differences in the qualitative composition and also in the quantity of individual components. Additionally, the analysis results strongly depend on the method used and the conditions of extraction and analysis of volatile compounds. The above discussion and attempt to compare previous studies of volatile compounds in RL confirm these observations and comments. To the best of our knowledge, there exist no studies on supercritical extracts from RL leaves. Hence, our results regarding the supercritical extract are difficult to compare with studies by other authors who analysed other types of extracts and other parts of the plant, additionally collected from other geographical locations. Studies conducted by Tasdemir et al. (2003) show some similarities in terms of extraction conditions (low temperature and low polarity of the solvents used, but during conventional extraction by maceration) and, subsequently, the use of the headspace GC-MS technique for identifying compounds. The supercritical leaf extracts obtained in this study (and previously reported also extracts from RL flowers) showed a greater variety of components and a higher content of monoterpenes (Tasdemir 2003; Łyko et al., 2024)These confirm our earlier observations and indicate the high efficiency of supercritical extraction in obtaining volatile compounds from RLL.

### 3.5 Biological activity of *Rhododendron luteum* leaf supercritical extracts

Terpenes and polyphenols are phytochemicals well known for their anti-inflammatory potential. It has been proven that methanolic extracts of RL leaves act against enzymes involved in the inflammatory process (Łyko et al., 2022). Therefore, it seemed reasonable to analyse the SC-CO_2_ extracts for their anti-inflammatory activity as well.

To assess the anti-inflammatory activity of the samples, the inhibition of pro-inflammatory enzymes was measured. Based on the Lineweaver-Burk curves using different concentrations of inhibitors and substrates, the mode of XO and LOX inhibition (competitive, non-competitive, or uncompetitive) was determined.

The few existing ethnobotanical data describe the use of *R. luteum* in traditional medicine as a remedy for rheumatoid diseases (Popescu and Kopp, 2013; Bussmann et al., 2020). Enzymes that play an important role in the inflammatory process in rheumatoid diseases are XO and LOX. Xanthine oxidase (XO) overactivity can lead to a common rheumatic disease: gout or acute arthritis. XO inhibition lowers blood uric acid concentrations and reduces vascular oxidative stress and inflammation (Kostic et al., 2015). In turn, lipoxygenase (LOX) is one of the enzymes that catalyse the conversion of fatty acids into active metabolites, which mediates the occurrence of inflammation. Thus, a substance with LOX or XO suppression activity could act as a potential anti-inflammatory agent (Hu and Ma, 2018).

**FIGURE 2:**
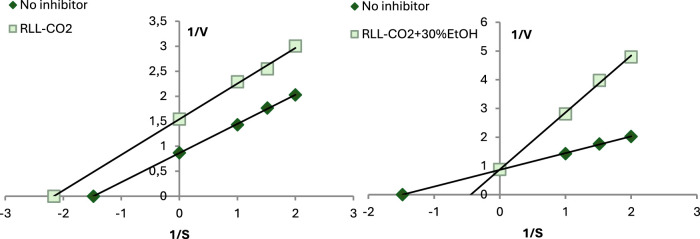
Mode of XO inhibitory activity of *R. luteum* leaf supercritical CO2 extracts: RLL-CO2; RLL-CO2+30%EtOH. Abbreviations as in Table 1.

As presented in Table 3, SC-CO2 extracts of *R. luteum* leaves varied significantly in their anti-XO activity. Expressed as IC_50_ values, the results were 2.11 ± 0.01 mg DE/mL for RLL-CO2+30%EtOH and 16.53 ± 0.05 mg DE/mL for RLL-CO2. The sample obtained with the addition of a modifier showed similar activity to oleanolic acid and to SC-CO2 flower extracts (Łyko et al., 2024). Conversely, the sample extracted by pure SC-CO2 was significantly less active. This is probably due to the much lower content of detected pentacyclic triterpenes and β-sitosterol. Another reason for the stronger inhibition of the enzyme by the RLL-CO2+30%EtOH extract may be the presence of more polar compounds from other groups, such as polyphenols, which were found to have high activity against XO in other studies (Hadj Salem et al., 2011; Mehmood et al., 2019). Moreover, there are reports of synergistic effects of the phytochemicals from the different groups, especially polyphenols (Lyu et al., 2019; Zhang et al., 2019; Guo et al., 2021). It was proven that they could regulate multiple pathways, cells, and inflammatory markers including proinflammatory enzymes. Besides, they can work in two ways each molecule individually can have specific interactions oninflammatory markers, or more phytochemicals may target the same inflammatory markers, e.g., luteolin present in the RLL-CO2+30%EtOH extract is known to interact in this way with other polyphenols (Zhang et al., 2019). The LC-MS analysis revealed the presence of phenolic acids that show anti-XO activity, e.g., salicylic acid and ferulic acids, which could inhibit XO with IC_50_ values of three and 5 μM, respectively (Hsu et al., 2021). Interestingly, RLL-CO2 showed an uncompetitive mode of action, while RLL-CO2+30%EtOH presented a competitive mode of XO inhibition (Figure 2). In turn, the triterpene standard–OA inhibited XO non-competitively.

**TABLE 3:** Anti-inflammatory (XO, LOX, hyaluronidase inhibitory effect) and antidiabetic (α-glucosidase and α-amylase inhibitory effect) activity demonstrated by *Rhododendron luteum* Sweet leaf supercritical extracts.

Sample	XO IC_50_ (mg DE/mL)	LOX IC_50_ (mg DE/mL)	Hyaluronidase IC_50_ (μg DE/mL)	α-glucosidase IC_50_ (μg DE/mL)	α-amylase (μg/mg of DE)
RLL-CO_2_	16.53 ± 0.05	0.57 ± 0.01	302.96 ± 25.15	89.36 ± 8.18	40.51 ± 1.16
RLLCO_2_+30%EtOH	2.11 ± 0.01	1.35 ± 0.05	113.45 ± 11.67	26.32 ± 0.31	602.84 ± 0.02
OA	1.87 ± 0.00	0.66 ± 0.04	Not active	27.93 ± 0.35	—
Allopurinol	0.03 ± 0.00	—	—	—	—
NDGA	—	2.58 ± 0.08	—	—	—
Acarbose	—	—	—	83.90 ± 3.33	—

Explanations: The results of xanthine oxidase (XO), lipoxygenase (LOX), hyaluronidase, and α-glucosidase inhibition are expressed as IC_50_ values; α-amylase inhibitory activity was expressed as acarbose equivalent per mg of dry extract. Abbreviations as in Table 1: DE, dry extract; OA, oleanolic acid; NDGA, nordihydroguaiaretic acid.

**FIGURE 3:**
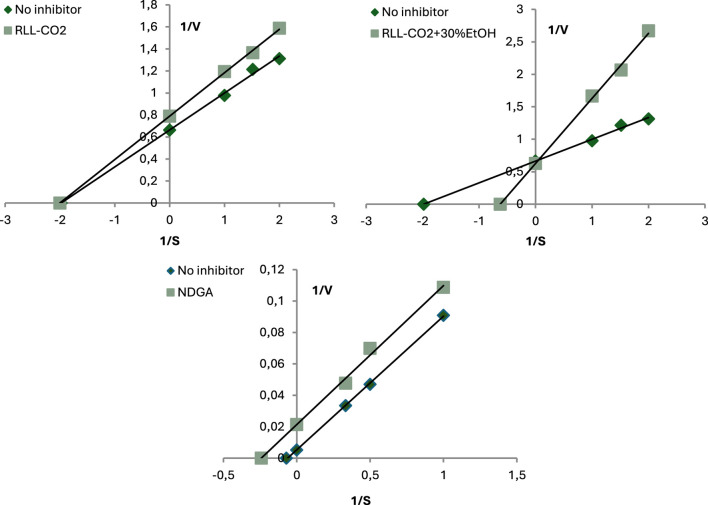
Mode of LOX inhibitory activity of *R. luteum* leaf supercritical CO2 extracts: RLL-CO2; RLL-CO2+30%EtOH; NDGA. Abbreviations as in Table 1.

Another enzyme included in the study of anti-inflammatory activity was lipoxygenase, as can be seen in Table 3. IC_50_ values of tested samples were similar to OA: 0.57 ± 0.01 mg DE/mL for RLL-CO2 and 1.35 ± 0.05 mg DE/mL for RLL-CO2+30%EtOH. Moreover, they were slightly less active than corresponding *R. luteum* flower SC-CO2 extracts (Łyko et al., 2024). Interestingly, the less-polar RLL-CO2 extract was found to be more active. This sample contained a lower concentration of the analysed triterpenes and probably fewer polyphenols than the polar sample. Nevertheless, a high content of volatile compounds was found in this extract. Among them were eugenol, limonene, and β-caryophyllene, previously designated as potent LOX inhibitors (Naidu, 1995; Baylac and Racine, 2003; Frum and Viljoen, 2006). RLL-CO2 presented the LOX inhibitory mode, defined as non-competitive (mixed inhibition), while RLL-CO2+30%EtOH showed the competitive inhibitory mode (Figure 3).

Hyaluronidase activity leads to the production of, e.g., tetrasaccharides, which are associated with the release of pro-inflammatory cytokines and angiogenesis. Therefore, one of the strategies for alleviating inflammation is hyaluronidase inhibition (Tekulu et al., 2020). The RL samples were proved to be the most active against hyaluronidase among all tested enzymes. The hyaluronidase inhibitory activity assay was based on the transmittance measurement of precipitated undigested hyaluronic acid. The tested samples inhibited the enzyme in a concentration-dependent manner. The IC_50_ values were 302.96 ± 25.15 for RLL-CO2 and 113.45 ± 11.67 mg DE/mL for RLL-CO2+30%EtOH. Oleanolic acid did not present any activity against hyaluronidase. As the main component of the extract showed no activity, it is presumed that the samples’ high activity is due to the presence of other active ingredients, such as other triterpenes or polyphenols. It is confirmed that adding ethanolic co-solvent in SC-CO2 extraction increases the elution of more polar compounds, e.g., flavonoids and phenolic acids. This would align with some previous studies that have proven the considerable capacity of polyphenols to block hyaluronidase (Gębalski et al., 2022). For instance, observed the most in RLL-CO2+30%EtOH, syringic acid was previously reported to inhibit hyaluronidase at IC_50_ value of 22.24 ± 2.27 μg/mL, showing that it is significantly more active than our samples (Maity et al., 2011). Other phenolic acids found in high amounts in the RLL-CO2+30%EtOH sample, i.e., ferulic acid and protocatechuic acid, were also proven to be potent hyaluronidase inhibitors with IC_50_ values of 396.12 μg/mL and 107.57 μg/mL, respectively (Girsang et al., 2020).

One of the ways to treat obesity and type 2 diabetes is to maintain adequate glucose levels. Lowering glucose levels can be achieved by inhibiting one of the carbohydrate-hydrolysing enzymes, such as α-amylase and α-glycosidase (de Sales et al., 2012). Many phytochemicals, such as phenolic acids, flavonoids, anthocyanins, carotenoids, alkaloids, sugars, proteins, fatty acids, and terpenes can block both of the abovementioned enzymes (Papoutsis et al., 2021). Accordingly, we decided to investigate the activity of terpene-rich RLL supercritical extracts against α-amylase and α-glycosidase.

As presented in Table 3., *R. luteum* leaf SC-CO2 samples presented a strong capacity to block α-glucosidase, demonstrating its potential efficiency in preventing glucose absorption from the digestive system. IC_50_ values of RLL-CO2+30%EtOH and OA were similar (26.32 ± 0.31 and 27.93 ± 0.35 μg DE/mL, respectively). This is in agreement with studies of OA’s effect on α-glycosidase activity. According to many scientific data, oleanolic and ursolic acids strongly inhibit the α-glucosidase in a dose-dependent manner (Castellano et al., 2013; 2016; Ding et al., 2018; Mioc, 2022a). These triterpenic acids, present in the sample in high abundance, may reduce post-prandial hyperglycemia, thereby helping control blood glucose levels and reducing oxidative stress, which is crucial in preventing insulin resistance and complications associated with type 2 diabetes. Kinetic studies suggest that these two molecules interact with α-glucosidase through a non-competitive inhibition mechanism (Castellano 2016; Mioc, 2022a). Moreover, many phenolic acids found in the more active RLL-CO2+30%EtOH demonstrated the capacity to inhibit α-glucosidase in some previous studies. According to the findings, the inhibitory capacity of ferulic acid was IC_50_ = 0.866 mg/mL (Zheng et al., 2020); for p-coumaric acid, IC_50_ = 1.02 mg/mL; for syringic acid, IC_50_ = 1.63 mg/mL and for protocatechuic IC_50_ = 0.14 mg/mL (Aleixandre et al., 2022). Moreover, as with the anti-inflammatory effect, there are some reports of synergistic effects of phytochemicals such as sesquiterpenes and flavonoids on α-glucosidase (Zeng et al., 2016; Fan et al., 2017). Among compounds detected in RLL-CO2+30%EtOH, luteolin and apigenin have been shown to have synergistic effects with other polyphenols on α-glucosidase (Zeng 2016; Hyeong-U et al., 2019). Our study has also shown that RLL-CO2+30%EtOH has a more potent inhibitory effect than acarbose. RLL-CO2 activity was slightly lower (89.36 ± 8.18 μg DE/mL) and comparable to the positive control (acarbose; 83.90 ± 3.33 μg DE/mL). Different extracts of aerial parts of *R. luteum* have been previously reported to inhibit α-glycosidase. In the mentioned study, acarbose equivalents were 24.19 and 25.02 mmol of acarbose/g of extract for ethyl acetate and methanol extract, respectively (Mahomoodally et al., 2020).

*R. luteum* SC-CO2 extracts show low anti-α-amylase activity compared to that of acarbose. This is in line with the abovementioned study on *R. luteum* (Mahomoodally et al., 2020). Interestingly, OA also exhibits significantly lower activity against α-amylase than α-glucosidase, aligning with some previous studies (Castellano et al., 2013). In the cited paper, the inhibitory activity of OA against amylase was IC_50_ = 0.1 mg/mL, and IC_50_ for α-glucosidase inhibition was 0.0046 mg/mL).

## 4 Conclusion

In this study, two-step supercritical CO_2_ extraction was used to obtain terpene-rich samples from *R. luteum* leaves. The application of detection methods such as HS-SPME-GC-FID/MS and LC-MS/MS allowed the thorough determination of the composition of the previously unexplored extracts prepared from this poorly explored plant material. It has been established that supercritical CO_2_ extraction with the addition of ethanol yields an extract with a high content of terpenes, especially oleanolic and ursolic acid, known for their antidiabetic and anti-inflammatory activity. Additionally, the polyphenol profile analysis also revealed the presence of syringic acid and prunetin, not previously detected in RL. Therefore, given also the numerous advantages of supercritical extraction, such as environmental friendliness and applicability on an industrial scale this type of extraction seems to be an excellent method for extracting bioactive metabolites from *R. luteum* leaves. Thus the optimization of the RL leaves SC-CO_2_ extraction would be crucial for the subsequent use of the industrial-scale extraction.

This is the first study to describe the capacity of RL leaf SC-CO2 extracts to inhibit enzymes related to inflammation (XO, LOX, hyaluronidase) and diabetes (α -amylase, α-glucosidase). The study revealed that the extracts are particularly effective inhibitors of α-glucosidase, an enzyme that plays an important role in carbohydrate digestion. By inhibiting this enzyme, RL’s phytochemicals can help control postprandial blood glucose levels, offering potential benefits in managing diabetes. However, while *in vitro* studies provide valuable preliminary insights, they skip very important aspects, such as bioavailability and metabolism criteria should be complemented by *in vivo* studies to ensure efficacy and safety of this action. This comprehensive examination supports the knowledge about the composition and health-promoting properties of *R. luteum* leaves, with promising implications for further applications of supercritical extracts in pharmacy, medicine, and cosmetology. The findings of this study are highly promising, providing a strong basis for further research, which will focus on optimizing supercritical extraction to maximize the yield of active compounds, scaling and standardization of the industrial extraction method, and further *in vitro* and *in vivo* preclinical studies, aimed at understanding the mechanisms of action and safety of use.

## Data Availability

The original contributions presented in the study are included in the article/Supplementary Material, further inquiries can be directed to the corresponding author.
