# Supplementary material for: Chemical profile of Rhododendron luteum Sweet leaf supercritical CO2 extracts and their anti-inflammatory and antidiabetic potential
Source: Front Chem. 2025 Apr 25;13:1576852. doi: 10.3389/fchem.2025.1576852 (PMC12062060; doi:10.3389/fchem.2025.1576852)
Supplement: Supplementary file 1 [file DataSheet1.pdf]

Supplementary Material

1 Supplementary Tables

Table S1. Summary of optimized parameters for the quantitative analysis of phenolic acids and flavonoids.

Compound	Retention time [min]	Precursor ion	Fragmentation ions	Collision energy (V)
Gallic acid	5.16	168.7	78.9	-36
			124.9	-14
3-Caffeoylquinic acid	6.93	352.9	191.1	-28
			178.9	-22
Protocatechuic acid	8.42	152.9	80.9	-26
			107.8	-38
5-Caffeoylquinic acid	9.24	353.0	190.9	-20
			85	-60
4-Caffeoylquinic acid	9.38	352.9	173	-21
			135	-36
			179	-22
4-Hydroxybenzoic acid	10.84	136.8	92.9	-18
Gentisic acid	11.37	152.8	80	-110
			96.9	-52
Caffeic acid	11.38	178.7	88.9	-46
			134.9	-16
Syringic acid	11.42	196.9	122.8	-24
			181.9	-12
3-Hydroxybenzoic acid	12.12	136.8	93	-16
			75	-48
4-Hydroxycinnamic acid (*p*-coumaric acid)	14.10	162.7	119	-14
			93	-44
Sinapic acid	14.47	222.8	121	-36
	14.94		148.9	-20
Ferulic acid	14.80	192.8	133.9	-16
	15.22		177.9	-12

3-Hydroxycinnamic acid (*m*-coumaric acid)	15.50	162.7	119	-14
			91	-36
Rosmarinic acid	15.91	358.7	160.8	-20
			196.8	-22
2-Hydroxycinnamic acid (*o*-coumaric acid)	16.80	162.7	119	-14
			93	-46
3,4-Dimethoxycinnamic acid	17.60	206.9	103	-16
			163	-12
Salicylic acid	17.91	136.8	93	-16
			75	-48
Catechin	9.64	288.8	244.9	-16
			109	-32
Epigallocatechin gallate (EGCG)	11.20	457	169.1	-30
			125	-30
Dihydromyricetin (Ampelopsin; Ampeloptin)	12.10	319	193	-30
			125	-30
Naringenin	14.52	270.8	119	-34
			150.9	-22
Taxifolin	15.15	302.7	124.9	-26
			284.8	-14
Myricetin	16.57	316.7	136.9	-32
			150.9	-26
Luteolin	17.82	284.7	132.9	-38
			150.9	-26
Eriodictiol	17.89	286.7	134.9	-32
			150.9	-18
Laricitrin (3'-O-Methylmyricetin)	17.9	330.97	151	-30
			315.9	-30
Quercetin	17.94	300.7	150.9	-26
			178.8	-20
3-*O*-Methylquercetin	18.11	314.7	299.8	-18
			270.8	-26
Apigenin	18.64	268.8	117	-44
			106.8	-34

Kaempferol	18.85	284.7	116.8<br>93	-46<br>-52
Isorhamnetin	18.99	314.7	299.7<br>150.9	-20<br>-30
Isokaempferide	19.16	298.8	283.9<br>226.9	-18<br>-28
Rhamnetin	20.10	314.7	165<br>120.9	-24<br>-36
Prunetin	21.98	282.8	267.7<br>238.7	-20<br>-26
Rhamnazin	22.37	328.7	270.8<br>313.8	-26<br>-14
Luteolin 3’,7’-diglucoside	11.28	609.1	285<br>447	-50<br>-32
Quercetin-3-*O*-rutinoside (Rutin)	11.99	608.7	299.6<br>270.9	-46<br>-60
Kempferol 3,7-dirhamnoside (Kaempferitrin)	12.16	576.8	284.8<br>430.9	-42<br>-30
Apigenin – 6-*C*-glucoside (Isovitexin)	12.38	430.8	310.9<br>340.9	-28<br>-26
Apigenin – 8-*C*-glucoside (Vitexin)	12.40	430.8	310.9<br>340.9	-26<br>-34
Quercetin-3-*O*-galactoside (Hyperoside)	12.80	462.7	299.7<br>254.7	-28<br>-42
Luteolin-7-*O*-glucoside (Luteoloside)	12.87	446.8	284.8<br>132.9	-30<br>-78
Quercetin-3-*O*-glucoside (Isoquercetin)	13.00	462.7	299.7<br>270.7	-30<br>-44
Naringenin-7-*O*-rutinoside (Narirutin)	13.80	578.9	270.8<br>118.9	-34<br>-76
Naringenin-7-*O*-rhamnosidoglucoside (Naringin)	14.50	579.1	151<br>271	-54<br>-42
Kaempferol – 3-*O*-glucoside (Astragalin)	14.66	446.7	226.8<br>254.8	-54<br>-40
Quercetin 3-*O*-rhamnoside (Quercitrin)	14.83	446.7	299.7<br>270.7	-30<br>-40

Apigenin 7-*O*-glucoside (Apigetrin, Cosmosin)	14.91	430.7	267.7	-38
			116.9	-84
Naringenin 7-*O*-glucoside	15.12	432.7	270.8	-22
			118.9	-64
Afzelin (Kaempferol 3-rhamnoside)	15.9	431.1	284.9	-30
			254.9	-30

Table S2. Analytical parameters used for quantitative determination of phenolic acids and flavonoids.

Compound	LOD [ng/mL]	LOQ [ng/mL]	R^2	Linearity range [ng/mL]
Gallic acid	1000	1850	0.9986	1850-18500
Protocatechuic acid	200	400	0.9988	1890-18900
5-Caffeoylquinic acid	75	180	0.9991	180-18000
Gentisic acid	82	205	0.9992	2050-19900
4-Hydroxycinnamic acid (*p*-coumaric acid)	83	200	0.9990	415-13800
Syringic acid	168	666	0.9993	666-11100
Sinapic acid	17.4	69.4	0.9999	69.4-3470
Ferulic acid	1250	1830	0.9985	1830-36500
Isoferulic acid	17.2	686	0.9997	343-11400
Salicylic acid	500	732	0.9974	1830-18300
Luteolin	6	16	0.9974	33-1650
Apigenin	15	22	0.9979	89-4470
Isorhamnetin	12	24	0.9975	40-60000
Prunetin	50	75	0.9985	200-20000
Hyperoside (Quercetin-3-*O*-galactoside)	167	250	0.9983	500-25000

Luteoloside (Luteolin-7-*O*-glucoside)	50	100	0.9980	250-25000
Apigetrin (Apigenin 7-*O*-glucoside)	100	250	0.9989	750-25000

Table S3. Volatile constituents of *R. luteum* leaf supercritical CO_2 extract identified by HS-SPME-GC-FID/MS.

**Constituent**	**RI_{exp}[a]**	**RI_{lit}[b]**	**RLL-CO_2 (%)**
Hexanal	783	785	1.43
Furfural	803	801	t
(*Z*)-Hex-3-en-1-ol	847	851	0.56
Hexanol	859	855	0.44
Heptanal	882	882	0.25
Benzaldehyde	932	936	0.43
α-Pinene	934	936	1.72
Camphene	945	944	0.13
Thuja-2,4(10)-diene	948	946	0.35
Oct-1-en-3-ol	962	962	0.32
6-Methylhept-5-en-2-one	970	972	0.38
β-Pinene	973	978	1.33
Hexanoic acid	984	983	1.36
(*E*,*E*)-Hepta-2,4-dienal	985	987	0.80
Myrcene	986	987	0.76
Decane	1000	1000	0.55
α-Phellandrene	1002	1002	0.31
Benzyl alcohol	1005	1006	2.33
p-Cymene	1013	1015	0.27
β-Phellandrene	1020	1023	0.28
1,8-Cineole	1021	1024	0.30
Limonene	1024	1025	13.10
(*E*)-Oct-2-en-1-al	1033	1034	0.30

(*E*)-β-Ocimene	1039	1041	0.12
(*E*,*Z*)-3,5-Octadien-2-one	1045	1050	0.13
γ-Terpinene	1050	1051	0.11
(*E*)-Oct-2-en-1-ol	1054	1055	0.11
Octanol	1058	1063	0.10
trans-Linalool oxide (f)	1060	1062	0.38
(*E*,*E*)-Octa-3,5-dien-2-one	1068	1070	0.33
Heptanoic acid	1071	1070	0.38
cis-Linalool oxide (f)	1074	1072	0.26
1-Acetyl-2-methylcyclopentene	1080	-	0.27
Nonanal	1084	1076	3.28
β-Phenylethanol	1086	1085	5.22
Undecane	1100	1100	0.45
α-Camphenal	1104	1103	0.19
Methyl octanoate	1107	1105	0.30
Camphor	1121	1123	1.00
trans-Pinocarveol	1125	1126	0.40
Menthone	1134	1136	0.55
Pinocarvone	1140	1137	0.44
Isomenthone	1144	1146	0.13
Ethyl benzoate	1149	1149	0.16
Borneol	1152	1150	0.47
cis-Linalool oxide (p)	1155	1148	0.12
p-Cymen-9-ol	1159	1157	0.45
Terpinen-4-ol	1163	1164	0.36
α-Terpineol	1174	1176	2.10
Estragole	1176	1179	t
Ethyl octanoate	1181	1177	1.75
Decanal	1185	1180	0.48

β-Cyclocitral	1196	1195	1.44
Dodecane	1199	1200	5.58
Bornyl formate	1207	1208	0.24
Carvone	1215	1214	2.24
Geraniol	1236	1235	0.33
Nonanoic acid	1258	1260	0.25
Thymol	1265	1267	t
Bornyl acetate	1270	1270	0.54
Carvacrol	1278	1278	0.10
Undecanal	1287	1286	0.14
Tridecane	1300	1300	2.90
Methyl decanoate	1306	1310	0.07
Eugenol	1331	1331	6.15
α-Cubebene	1350	1355	0.07
Methyleugenol	1365	1369	0.14
α-Ylangene	1371	1376	0.14
α -Copaene	1377	1379	0.21
Ethyl decanoate	1376	1375	0.69
β-Bourbonene	1385	1387	0.08
Tetradec-1-ene	1389	1387	0.47
Tetradecane	1400	1400	2.20
α-Ionone	1406	1407	0.47
α-Gurjunene	1411	1413	0.16
β-Caryophyllene	1419	1420	3.91
Geranylacetone	1429	1430	1.12
Calarene	1434	1437	3.17
Aromadendrene	1440	1443	0.10
Selina-4(15),6-diene	1447	1449	0.16
α-Humulene	1452	1455	0.45

Dodecanol	1459	1460	3.05
β-Ionone epoxide	1462	1460	1.06
β-Ionone	1465	1467	3.03
γ-Muurolene	1472	1474	0.23
Eugenol acetate	1485	1483	0.55
Dihydroactinidiolide	1491	1493	3.82
α-Selinene	1492	1494	0.45
α-Muurolene	1494	1496	t
Pentadecane	1550	1500	0.21
γ-Cadinene	1507	1507	0.47
cis-Calamenene	1510	1517	0.07
δ-Cadinene	1515	1520	0.83
Cadina-1,4-diene		1523	t
α-Calacorene	1530	1527	0.10
Spathulenol	1565	1572	0.07
Caryophyllene oxide	1572	1578	0.14
Ethyl dodecanoate	1576	1579	0.07
Unknown	1583	-	0.23
Hexadecane	1597	1600	0.18
α-Cadinol	1639	1642	t
Total identified			95.82

[a] – experimental retention indices on the nonpolar column; b – literature retention indices according to MassFinder and NIST libraries; RLL-CO_2 – *R. luteum* leaf supercritical extract;- – not detected; t – trace, <0.04%; Unknown (RI 1583), EIMS 70eV, m/z (%): 161(100), 93(75), 43(47), 123(41),121 (40), 204(39), 189(31), 81(30), 105(29), 119(28), 91(27), 69(23), 95(23), 107(23), M 222(2), EIMS 70eV, m/z (%): 161(100), 43(59),93(57),121(56),119(42), 163(37), 105(32), 91(29), 81(27), 120(27), 107(26), 95(25), 189(24), 69(22), M 222(2).
